# Pediatric residents' knowledge of epidemiology and statistics

**DOI:** 10.5116/ijme.5c01.628f

**Published:** 2018-12-21

**Authors:** Darnna Banks, Pamela Botchway, Simi Akintorin, Rosibel Arcia, Kenneth Soyemi

**Affiliations:** 1 Departments of Pediatrics, Cook County Health, and Hospitals System, John H Stroger Jnr Hospital, Chicago, Illinois, USA; 2 Keck School of Medicine of USC, Medical School, Los Angeles, California, USA

**Keywords:** Pediatric, residents' knowledge, epidemiology and statistics, USA

## To the Editor

With access to the information via digital and hardware platforms and formats, residents (postgraduate trainees) have access to millions of published medical articles which can be found on a variety of electronic databases. Resident physicians and clinicians generally depend on the medical literature to keep their clinical knowledge up to date and apply evidence-based methods (EBM) to answer specific clinical questions. Although knowledge of epidemiology and statistics (E&S) is essential for comprehending medical evidence, research has shown consistently low and variable knowledge among resident physicians. Simultaneously, the complexity of statistical methods reported in top-tier medical journals continues to be a challenge, as it makes the journals difficult to understand.^1^^, ^^2^ A study showed that the majority of the responding residents agreed that research is essential and improves health care and that it helps in building a future academic career. Lack of research training, lack of time, work-related stress, and lack of supervisors are perceived barriers to doing research.^3^ Teaching methods of E&S are wide-ranging; examples include blended format, case studies, didactic lectures, computer simulations, etc.^4^ There are few published papers about residents' ability to understand statistical methods or how to interpret research outcomes appropriately. We have noticed that during our journal club meetings, medical residents either skip the statistical methods or describe results without fully understanding the important aspects of the statistical analysis. We surveyed with the aim of describing residents understanding of E&S concepts used in the interpretation of research results and describe the need for additional E&S training to help medical residents evaluate and translate research into clinical practice. The survey was made up of 19 multiple-choice E&S knowledge questions, 3 demographic questions, and 1 Likert scale question which addressed residents' desire to receive more E&S teaching. The question regarding the desire to learn more E&S was rated on a 5-point Likert scale, in which 1 represented strongly disagree, and 5 represented strongly agree. The survey was completed by 79% (n=82) residents, among whom 62% were interns (first-year resident), 78% were female, 95% were international medical graduates (physicians who received their medical school education outside the United States or Canada). The vast majority of participants agreed that more training opportunities regarding E&S and its application to medical sciences are needed. On individual knowledge questions, across all levels of training, the most correctly answered question was related to the recognition of the value of masking in studies to prevent bias. This was answered correctly by the vast majority of the participants. Few medical residents correctly responded to the question regarding Cox's proportional hazard assessment, and the use and interpretation of an ANOVA test. Across all levels of training, there was variable performance (i.e., percent correct responses) in basic E&S concepts. In basic concepts areas, where we expected high performance, we saw the opposite (examples include the identification of continuous variables, interpretation of standard deviation (SD), applied analysis using the Chi-Square test and ANOVA); surprisingly, in the complex areas, such as randomization, the percentage giving a correct score was high across all levels of training, and this was similar to the findings of a previous study.^5^ The reason for the discordance is not known but may be attributed to differences in residents' medical school training, knowledge base regarding E&S, participation in advanced training, mathematical knowledge, and interest in statistics^6^ that most participants answered correctly. Studies continue to show clinicians and residents have difficulty in understanding and interpreting p-values.^7^ Analysis showed that residents with previous advanced degrees generally fared better on E&S knowledge tests, supporting the advantages of having advanced training linking with ease in the interpretation of applied E&S concepts included in medical journals.^8^ A previous study that described residents' E&S attitudes found that both the attitudes and confidence of residents were positively associated with the number of journals read monthly and formal coursework in biostatistics.^9^ Therefore it would be helpful to encourage  residents to improve their attendance and active participation in the monthly journal clubs. Most of the residents expressed an interest in more E&S teaching, which would be a gateway to the better interpretation of journal articles and application of EBM methods. Also, our study was limited by the small sample size allowing prior preparation or forewarning the residents may have improved some of the test scores. Scores were not influenced by gender or level of training. The universal need for additional training was identified and will be addressed using blended methods of online training, teaching, participation in a journal club, and the sharing of current statistical methods as they are published in the literature. Residents and students continue to articulate the relevance of E&S to real clinical issues. Previously  published work had suggested that the causes of poor interest in E&S was due to the short duration of E&S teaching in the medical curriculum, the lack of practice exercises, and the need for practical data collection.^10^ Even though we did not study the barriers, we intend to extrapolate what is known in the literature to mitigate and plan a custom-made curriculum that works best for current and future residents. Our survey showed some residents had trouble with correctly answering simple and complex E&S concepts; we can align this with our continued observations of similar difficulty during our monthly journal club and scholarly activities. We continue to observe that some of our residents still have difficulties interpreting research methodology and statistical details, even though it is expected that a medical school training would have prepared them to analyze basic biomedical data using simple statistical methods or collaborating with a biostatistician in cases that need more complex methods. In conclusion, additional studies to describe specific E&S training needs that will help residents rapidly evaluate and translate research into clinical practice are needed.

### Conflict of Interest

The authors declare that they have no conflict of interest.

## References

[1] Barlow PB, Skolits G, Heidel RE, Metheny W, Smith TL (2015). Development of the Biostatistics and Clinical Epidemiology Skills (BACES) assessment for medical residents.. Postgrad Med J.

[2] Best AM, Laskin DM (2013). Oral and maxillofacial surgery residents have poor understanding of biostatistics.. J Oral Maxillofac Surg.

[3] Mitwalli HA, Al Ghamdi KM, Moussa NA (2014). Perceptions, attitudes, and practices towards research among resident physicians in training in Saudi Arabia.. East Mediterr Health J.

[4] Rahman M, Chowdhury M, Ahmad MM, Fukui T (2000). Do taught courses on community medicine change knowledge status regarding clinical epidemiology and biostatistics in medical students?. J Epidemiol.

[5] Polychronopoulou A, Eliades T, Taoufik K, Papadopoulos MA, Athanasiou AE (2011). Knowledge of European orthodontic postgraduate students on biostatistics.. Eur J Orthod.

[6] Zeimet R, Kreienbrock L, Doherr MG (2015). Teaching biostatistics and epidemiology in the veterinary curriculum: what do our fellow lecturers expect?. J Vet Med Educ.

[7] Wellek S (2017). A critical evaluation of the current "p-value controversy".. Biom J.

[8] Windish DM, Huot SJ, Green ML (2007). Medicine residents' understanding of the biostatistics and results in the medical literature.. JAMA.

[9] Susarla SM, Lifchez SD, Losee J, Hultman CS, Redett RJ (2016). Plastic Surgery Residents' Understanding and Attitudes Toward Biostatistics: A National Survey.. Ann Plast Surg.

[10] Daher AM, Amin F (2010). Assessing the perceptions of a biostatistics and epidemiology module: views of Year 2 medical students from a Malaysian university. A cross-sectional survey.. BMC Med Educ.

